# Age- and Sex-Specific Distribution of the Triglyceride-Glucose Index in a Large Chinese Population: Cross-Sectional Study

**DOI:** 10.2196/95855

**Published:** 2026-05-27

**Authors:** Hanze Du, Shuaihua Song, Xiao Zhai, Junchi Liu, Yue Jiang, Yaoda Hu, Huijing He, Daowei Li, Huijuan Zhu, Shi Chen, Guangliang Shan, Hui Pan

**Affiliations:** 1 Key Laboratory of Endocrinology of National Health Commission, Department of Endocrinology, State Key Laboratory of Complex Severe and Rare Diseases, Peking Union Medical College Hospital, Chinese Academy of Medical Sciences & Peking Union Medical College, No. 1 Shuaifu Garden, Beijing, China, 86 (010) 69155685; 2 Department of Epidemiology and Statistics, Institute of Basic Medical Sciences, Chinese Academy of Medical Sciences & School of Basic Medicine, Peking Union Medical College, Beijing, China; 3 State Key Laboratory of Common Mechanism Research for Major Diseases, Beijing, China; 4 Jilin Provincial Key Laboratory of Tooth Development and Bone Remodeling, School and Hospital of Stomatology, Jilin University, Changchun, Jilin, China

**Keywords:** triglyceride-glucose index, age- and sex-specific distribution, glucolipid metabolism, china national health survey, chinese adults

## Abstract

**Background:**

The triglyceride-glucose (TyG) index has demonstrated promising predictive capability in clinical studies, but its distribution characteristics across different age and sex groups in the Chinese population have not been fully characterized.

**Objective:**

This study aimed to describe the population-based distribution of the TyG index.

**Methods:**

A total of 4621 participants aged 20‐80 years from the China National Health Survey were included in this study. The TyG index was calculated from fasting blood glucose and triglycerides. The age- and sex-specific distribution values of the TyG index were obtained using the percentiles method.

**Results:**

Males had higher BMI (23.32, SD 2.63 vs 22.72, SD 2.60 kg/m^2^), triglyceride (133.78, SD 78.07 vs 111.58, SD 61.18 mg/dL), fasting glucose (97.05, SD 11.95 vs 94.34, SD 10.46 mg/dL), and TyG index values (8.63, SD 0.54 vs 8.45, SD 0.50) than females. The TyG index of males reached its peak value at approximately 40 years of age. The lower limit percentile values for females exceeded that of males around age 50. After the age of 60, the upper limit of the distribution values in females was higher than in males.

**Conclusions:**

This study characterized the age- and sex-specific distribution of the TyG index among Chinese adults aged 20‐80 years. The results of this study contribute to a more precise assessment of glucolipid metabolism.

## Introduction

Metabolic syndrome has emerged as a major global health challenge, characterized primarily by insulin resistance, hyperglycemia, dyslipidemia, abdominal obesity, and hypertension [1]. These components are not only significant risk factors for chronic conditions such as cardiovascular disease, but also contribute to increased mortality rates [2]. Early detection and intervention for insulin resistance, a core feature of metabolic syndrome, are crucial to slowing disease progression [3]. However, traditional methods for assessing insulin resistance, such as the Homeostasis Model Assessment of Insulin Resistance, often require complex laboratory testing and incur high costs [4]. Consequently, the exploration of simple and cost-effective indicators to evaluate insulin resistance in clinical practice is of great importance.

In recent years, the triglyceride-glucose index (TyG index) has gained attention for its ability to effectively estimate insulin resistance. The TyG index is a simple, economical screening tool for this purpose [5-7]. Studies have shown that the TyG index is closely associated with metabolic syndrome, diabetes, cardiovascular disease, and mortality and has the potential to become a next-generation predictor for these conditions [4,8,9].

Although the TyG index has demonstrated promising predictive capability in clinical studies, its distribution characteristics across different age and sex groups have not been fully characterized in the Chinese population. Consequently, understanding population-based distribution patterns of the TyG index may improve interpretation of this marker in different demographic groups. This study aims to characterize the age- and sex-specific distribution of the TyG index in Han Chinese adults, thereby providing population-based distribution data for future research and clinical assessments of glucolipid metabolism.

## Methods

### Data Source and Study Population

Data for this study were obtained from the China National Health Survey database and included participants who met the criteria in Guangdong, Jilin, and Jiangsu provinces [10]. Exclusion criteria included (1) age <20 years or >80 years; (2) non-Han ethnicity; (3) BMI <14 kg/m^2^ or >28 kg/m^2^ [11]; (4) missing data for fasting glucose (GLU) or triglyceride (TG); and (5) participants with diabetes mellitus, hypertension, hyperlipidemia, hyperuricemia, other cardiovascular and cerebrovascular diseases, hepatic and renal diseases, thyroid disorders, pancreatic disorders, tumors, and other acute diseases. Information on disease history was assessed based on self-reports using standardized questionnaires. A total of 4621 participants were included in this study.

### Data Collection

All participants were required to fast for at least 8 hours, after which fasting blood samples were collected the following morning by professional nurses. The separated plasma or serum was aliquoted before the initial thawing and stored at −80°C to ensure sample stability. Serum TG and GLU levels were measured using a chemical analyzer (Cholesterol Cobas8000C701), and TyG index values were calculated using the formula TyG index=ln(TG [mg/dL] × GLU [mg/dL] / 2) [12]. Additionally, demographic information, including birth date, gender, and history of diabetes, hypertension, hyperlipidemia, hyperuricemia, and other relevant medical conditions, was collected through standardized questionnaires, ensuring the comprehensiveness and accuracy of the data.

### Ethical Considerations

This study was conducted according to the principles of the Declaration of Helsinki and approved by the institutional review board of the Institute of Basic Medical Sciences, Chinese Academy of Medical Sciences (number 028‐2013). All participants provided their consent for participation. Participants were informed that their personal information and research data would be kept strictly confidential, securely managed, and used only for research purposes in accordance with the approved study protocol and relevant ethical requirements.

### Statistical Analysis

Continuous variables were presented as mean (SD). One-way ANOVA was performed for intergroup comparisons, followed by post hoc multiple comparisons using the Bonferroni method. The age- and sex-specific percentiles (2.5th, 50th, and 97.5th) of the TyG index were calculated directly from the empirical distribution (ie, the ordered values) within each subgroup. The 95% CIs for these percentiles were estimated using the binomial exact method, a robust nonparametric approach. To visualize the age- and sex-specific distribution ranges for the TyG index, we constructed grouped percentile curves. Specifically, after stratifying the sample by sex and 10-year age intervals, we calculated the 2.5th percentile (lower limit) and the 97.5th percentile (upper limit) of the TyG index within each group. The curves were generated by smoothing splines through the midpoints of successive age groups to depict the trend. The scatter points illustrate the distribution of the individual raw data. All statistical analyses were conducted using Stata (version 16.0) and R (version 4.3.1) software, with the “tidyverse” and “ggplot2” packages used for data manipulation and visualization. A 2-sided significance determined as *P*<.05.

## Results

**Figure 1.**
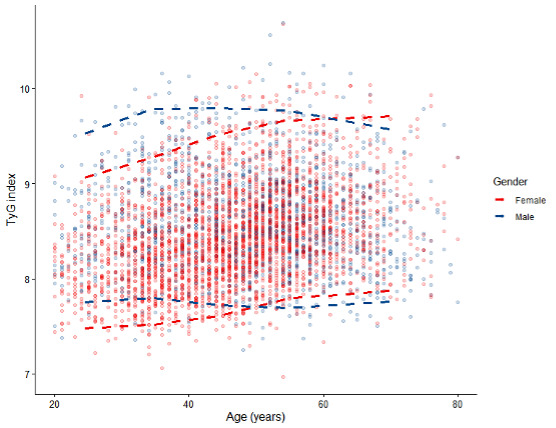
The distribution (2.5th-97.5th percentile) of TyG index values by age and sex. TyG: triglyceride-glucose.

**Figure 2.**
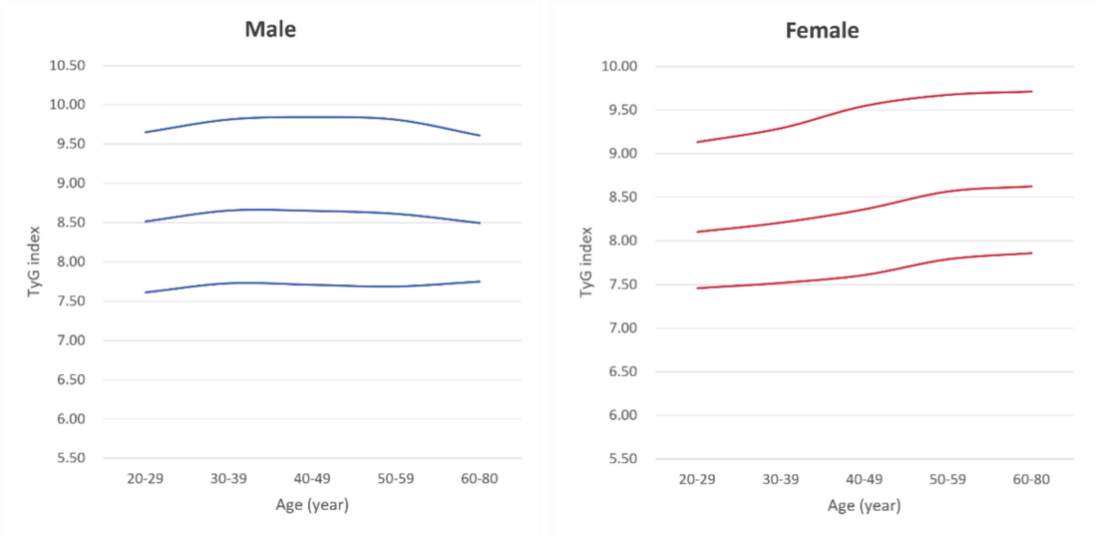
The age- and sex-specific distribution of TyG index values. TyG: triglyceride-glucose.

A total of 4621 subjects were included in this study, with a total of 1418 males and 3203 females. More baseline characteristics are shown in Table S1 in Multimedia Appendix 1. Figure 1 shows the age- and sex-specific distribution (2.5th to 97.5th percentiles) of the TyG index values. Table 1 and Figure 2 illustrate the age- and sex-specific percentile values and distribution of TyG index values. In this study, the TyG index values of females exhibited a gradual increase with age. In contrast, in males, the TyG index values peaked around 40 years of age and subsequently declined. The lower percentile value of the TyG index for females surpassed that for males at approximately 50 years of age. Furthermore, from 20 to 59 years, females showed lower upper percentile values of the TyG index compared with males. After 60 years of age, the upper percentile values of the TyG index were higher in females than in males. We also analyzed age- and sex-specific TyG index values across the three provinces, with detailed results provided in Table S2 in Multimedia Appendix 2.

**Table 1.** Distribution of triglyceride-glucose index values by age and sex.

	Number of participants	2.5% (95% CIs)	50.0% (95% CIs)	97.5% (95% CIs)
Males, age groups (years)				
20‐29	115	7.61 (7.38‐7.88)	8.52 (8.35‐8.63)	9.65 (9.28‐9.81)
30‐39	261	7.73 (7.45‐7.86)	8.65 (8.55‐8.74)	9.82 (9.70‐10.03)
40‐49	316	7.71 (7.53‐7.80)	8.65 (8.56‐8.72)	9.84 (9.69‐9.98)
50‐59	403	7.69 (7.59‐7.73)	8.61 (8.54‐8.68)	9.81 (9.64‐10.16)
60‐80	323	7.75 (7.59‐7.82)	8.50 (8.42‐8.57)	9.61 (9.49‐9.99)
Females, age groups (years)				
20‐29	251	7.46 (7.36‐7.54)	8.10 (8.04‐8.19)	9.13 (8.90‐9.53)
30‐39	665	7.52 (7.47‐7.57)	8.21 (8.15‐8.25)	9.29 (9.17‐9.35)
40‐49	951	7.61 (7.57‐7.64)	8.36 (8.32‐8.40)	9.55 (9.41‐9.63)
50‐59	912	7.79 (7.73‐7.83)	8.57 (8.54‐8.61)	9.68 (9.61‐9.84)
60‐80	424	7.86 (7.79‐7.93)	8.63 (8.55‐8.70)	9.71 (9.61‐9.93)

## Discussion

### Principal Findings

This study established age- and sex-specific percentile distributions of the TyG index based on 4621 Han Chinese adults. To our best knowledge, this is the first study to characterize age- and sex-specific distribution patterns of TyG index values in a large Chinese population. These findings provide population-based distribution data that may support the interpretation of TyG index levels in future epidemiological and clinical research.

The TyG index has been widely used in the diagnosis and management of various diseases, including cardiovascular and cerebrovascular diseases, metabolic diseases, and liver and kidney diseases [4]. However, the population-based distribution characteristics of the TyG index in apparently healthy individuals remain incompletely characterized. This study found that males had higher BMI, TG, GLU, and TyG index values compared to females, which was consistent with previous research [13-15]. The male participants in this study were slightly older than the females. Given that metabolic indicators such as TG and GLU are positively correlated with age, the higher TyG index values observed in males may be partly attributable to this age difference [16]. Moreover, studies have indicated potential gender differences in metabolic processes, particularly in glucolipid metabolism. These differences may be associated with the modulatory effects of sex hormones on lipid and glucose homeostasis [17].

Subsequently, we analyzed the TyG index values of participants from three different provinces in China by age and gender, and the results showed that healthy males aged 60‐80 years from Jilin had higher TyG index values than the others. These differences are likely driven by a combination of geographic, economic, lifestyle, and dietary factors. First, at the geographic and climatic level, the three provinces are listed in a north-to-south geographic order: northeastern China (Jilin), eastern China (Jiangsu), and southern China (Guangdong), respectively, with significant differences in latitude, mean annual temperature, and humidity. These variations may indirectly influence glucolipid metabolism by affecting basal metabolic rates, seasonal food availability, and physical activity patterns [18]. Second, socioeconomic and urbanization factors differ across the three provinces in terms of economic development, urbanization rates, and health care accessibility, which may act as potential confounders. Third, dietary factors and environmental conditions may also play a role. Jilin Province, located in the northeastern part of China, experiences long and harsh winters, and the local dietary pattern may be characterized by relatively high energy intake. These factors could potentially contribute to differences in metabolic profiles across regions. Particularly in the older adult population, although participants in this region do not report metabolic-related diseases, age-related decline in metabolic function may be associated with gradual increases in blood lipid and glucose levels [19]. Therefore, the observed differences should be interpreted as the result of synergistic effects of multiple environmental and lifestyle factors. However, these explanations remain speculative, as dietary intake and environmental exposure were not directly measured in the present study. Future studies should collect more comprehensive individual-level data on socioenvironmental and behavioral factors to more accurately elucidate the causes of cardiometabolic risk differences across various regions of China.

Our findings indicated that the median TyG index values peaked in males aged 40 to 49 years. In males older than 50 years, blood glucose levels were slightly elevated and triglyceride levels were significantly lower; consequently, the TyG index values were gradually surpassed by those of females. Although men generally have a higher cardiometabolic risk than premenopausal women, the lower TyG index values observed among males over 50 years in this study may be partly associated with healthier lifestyle and dietary habits among these participants [20,21]. Our results indicate that among individuals aged 50 and above, compared to females, males may need to maintain their TyG index values within a stricter range to sustain a relatively healthy metabolic state. In females, TyG index values increased with age. Furthermore, sex differences in the TyG index distribution varied with age. After approximately 50 years of age, females showed higher lower percentile values than males, and by around 60 years, females also exhibited higher upper percentile values. Current studies have demonstrated that postmenopausal women are at an increased risk of insulin resistance and glucose intolerance, accompanied by a gradual elevation in lipid levels [22,23]. Estrogen plays a crucial regulatory role in glucolipid metabolism, and the abrupt decline in estrogen levels following menopause significantly elevates the risk of metabolic syndrome and cardiovascular diseases [24,25]. Nevertheless, females were generally able to maintain a relatively favorable metabolic condition even at higher TyG index values.

Recent studies have increasingly focused on the association between TyG index thresholds and disease-specific morbidity or mortality risks. A study by Zhang et al [26], which used the National Health and Nutrition Examination Survey database, demonstrated that baseline TyG index values exceeding specific thresholds (TyG index >9.05 for all-cause mortality and >8.84 for cardiovascular mortality) exhibited positive correlations with mortality risk. Similarly, Liu et al [27] identified TyG index inflection points at 9.104 for all-cause mortality and 8.758 for cardiovascular mortality. Zhou et al [28] further found TyG index thresholds (male >8.81, female >8.73) as indicators of insulin resistance in Chinese populations. Notably, the upper percentile values of the TyG index observed in the study population were higher than previously reported cutoff values. This discrepancy may be partly attributed to differences in population characteristics and study design, including variations in inclusion and exclusion criteria across studies. Importantly, previously reported cutoff values were primarily derived from prognostic or risk prediction models for cardiometabolic outcomes, where thresholds are optimized to enhance predictive performance rather than to reflect population-based distribution characteristics. In addition, given the nature of large-scale population-based surveys, our study population may inevitably include individuals with undiagnosed or subclinical metabolic abnormalities. Moreover, to better reflect real-world population structure, individuals with overweight were included, which may also contribute to the observed differences in distribution compared with previously reported thresholds. These findings highlight the importance of characterizing population-specific distribution patterns of the TyG index to improve the interpretation of physiological variation across different demographic groups.

This study has several strengths. First, the inclusion of 4621 participants ensured a sample size that was moderate and statistically significant. Additionally, participants recruited from geographically diverse and representative provinces across China enhanced the reliability of the findings. Limitations should also be acknowledged. The assessment of disease history in this study was primarily based on participant self-reports. While this method serves as a common operational definition in large-scale epidemiological investigations, it may not fully exclude individuals with undiagnosed or subclinical conditions. Due to the limited sample size in the age 70‐80 subgroup, individuals aged 60‐80 were combined into a single stratum for analysis. While this ensures more statistically stable percentile estimates, it may obscure important physiological trends within this broad 20-year age span, given the considerable heterogeneity in metabolic parameters among older adults. The precision of the estimated percentile values may be limited in some age-sex subgroups with smaller sample sizes. Furthermore, the distribution characteristics in this study are derived from Han Chinese populations in three provinces—Jilin, Jiangsu, and Guangdong—listed in north-to-south geographic order and representing northeastern, eastern, and southern China, respectively. While the results reflect the characteristics of the Han Chinese in these regions, caution should be exercised when extrapolating them to other geographical areas or ethnic groups across the nation. Further studies are needed to characterize the age- and sex-specific distribution of the TyG index in other ethnic groups and in younger populations, including adolescents.

### Conclusion

This study characterized the age- and sex-specific distribution of TyG index values in Chinese adults aged 20‐80 years using a large national survey. These population-based distribution data provide a reference framework for future epidemiological and clinical research on glucolipid metabolism.

## Supplementary material

10.2196/95855 Multimedia Appendix 1 Characteristics of the study population.

10.2196/95855 Multimedia Appendix 2 Distribution of triglyceride-glucose index by age and sex in three provinces.

## Abbreviations

GLU: fasting glucose
TG: triglyceride
TyG: triglyceride-glucose
