# Supplementary material for: Age- and Sex-Specific Distribution of the Triglyceride-Glucose Index in a Large Chinese Population: Cross-Sectional Study
Source: JMIR Diabetes. 2026 May 27;11:e95855. doi: 10.2196/95855 (PMC13215049; doi:10.2196/95855)
Supplement: Multimedia Appendix 1 [file diabetes-v11-e95855-s001.docx]

**Multimedia Appendix Table 1 Characteristics of the Study Population**

|  |  | **Guangdong** | **Jiangsu** | **Jilin** |
| --- | --- | --- | --- | --- |
| **Male** |  |  |  |  |
| N | 1418 | 274 | 593 | 551 |
| Age (year) | 48.93 ± 13.17 | 50.77±14.13^a^ | 49.81±13.43^a^ | 47.07±12.16^b^ |
| BMI (kg/m2) | 23.32 ± 2.63 | 23.35±2.55^a,b^ | 23.55±2.56^a^ | 23.05±2.72^b^ |
| TG (mg/dl) | 133.78 ± 78.07 | 131.92±76.62 | 130.79±77.06 | 137.92±79.80 |
| GLU (mg/dl) | 97.05 ± 11.95 | 94.10±11.14^a^ | 97.29±12.03^b^ | 98.25±12.02^b^ |
| TyG index | 8.63 ± 0.54 | 8.58±0.54 | 8.62±0.52 | 8.68±0.55 |
| **Female** |  |  |  |  |
| N | 3203 | 697 | 1358 | 1148 |
| Age (year) | 46.46 ± 11.63 | 47.19±11.88^a^ | 47.19±11.90^a^ | 44.90±10.97^b^ |
| BMI (kg/m2) | 22.72 ± 2.60 | 22.90±2.55^a^ | 23.04±2.53^a^ | 22.24±2.63^b^ |
| TG (mg/dl) | 111.58 ± 61.18 | 119.22±67.17^a^ | 108.55±57.60^b^ | 110.54±61.14^b^ |
| GLU (mg/dl) | 94.34 ± 10.46 | 92.47±10.39^a^ | 95.95±10.37^b^ | 95.96±10.37^c^ |
| TyG index | 8.45 ± 0.50 | 8.49±0.50^a^ | 8.42±0.49^b^ | 8.45±0.51^c^ |

Data were presented as mean ± standard deviation (SD).

Different superscript indicated statistically significant differences between groups (*P* < 0.05, *P* value was corrected by Bonferroni method).

Abbreviations: TyG, triglyceride-glucose; BMI, body mass index.
