# Supplementary material for: Age- and Sex-Specific Distribution of the Triglyceride-Glucose Index in a Large Chinese Population: Cross-Sectional Study
Source: JMIR Diabetes. 2026 May 27;11:e95855. doi: 10.2196/95855 (PMC13215049; doi:10.2196/95855)
Supplement: Multimedia Appendix 2 [file diabetes-v11-e95855-s002.docx]

**Multimedia Appendix Table 2 Distribution of Triglyceride-Glucose Index by Age and Sex in Three Provinces**

|  | **Guangdong** | | |  |
| --- | --- | --- | --- | --- |
| **Male** | **N** | **TG(mg/dl)** | **GLU(mg/dl)** | **TyG index** |
| Age groups |  |  |  |  |
| 20-29 | 27 | 123.01±70.61 | 88.01±8.27 | 8.45±0.56 |
| 30-39 | 36 | 139.74±65.67 | 90.95±9.58 | 8.64±0.51 |
| 40-49 | 49 | 157.98±94.21 | 96.48±12.02 | 8.77±0.62 |
| 50-59 | 83 | 127.52±73.62 | 94.77±12.44 | 8.56±0.51 |
| 60-80 | 79 | 119.85±71.70 | 95.43±9.76 | 8.51±0.52^a,b^ |
|  | **Jiangsu** | | |  |
| **Male** | **N** | **TG(mg/dl)** | **GLU(mg/dl)** | **TyG index** |
| Age groups |  |  |  |  |
| 20-29 | 43 | 113.29±51.08 | 93.78±14.46 | 8.49±0.44 |
| 30-39 | 111 | 152.09±92.39 | 92.68±7.76 | 8.70±0.56 |
| 40-49 | 126 | 141.85±79.90 | 95.79±8.39 | 8.69±0.52 |
| 50-59 | 148 | 132.62±77.61 | 98.95±11.97 | 8.65±0.52 |
| 60-80 | 165 | 110.95±62.44 | 100.96±14.53 | 8.51±0.50^a^ |
|  | **Jilin** | | |  |
| **Male** | **N** | **TG(mg/dl)** | **GLU(mg/dl)** | **TyG index** |
| Age groups |  |  |  |  |
| 20-29 | 45 | 134.82±60.51 | 90.59±5.99 | 8.62±0.46 |
| 30-39 | 114 | 155.93±91.44 | 95.19±7.89 | 8.76±0.57 |
| 40-49 | 141 | 136.06±79.76 | 96.37±10.23 | 8.64±0.56 |
| 50-59 | 172 | 132.80±80.09 | 101.44±14.12 | 8.67±0.56 |
| 60-80 | 79 | 128.15±67.87 | 103.49±13.38 | 8.68±0.48^b^ |
|  | **Guangdong** | | |  |
| **Female** | **N** | **TG(mg/dl)** | **GLU(mg/dl)** | **TyG index** |
| Age groups |  |  |  |  |
| 20-29 | 56 | 101.70±64.05 | 87.56±7.07 | 8.28±0.44^a^ |
| 30-39 | 119 | 93.93±39.76 | 88.84±7.32 | 8.25±0.42 |
| 40-49 | 187 | 118.79±75.14 | 91.13±8.74 | 8.45±0.55 |
| 50-59 | 229 | 130.15±67.10 | 93.70±10.07 | 8.61±0.46 |
| 60-80 | 106 | 134.02±69.55 | 98.81±14.10 | 8.68±0.47 |
|  | **Jiangsu** | | |  |
| **Female** | **N** | **TG(mg/dl)** | **GLU(mg/dl)** | **TyG index** |
| Age groups |  |  |  |  |
| 20-29 | 114 | 78.39±34.86 | 87.42±6.50 | 8.06±0.39^b^ |
| 30-39 | 246 | 90.77±43.31 | 90.54±8.92 | 8.22±0.46 |
| 40-49 | 386 | 102.59±46.36 | 93.41±9.05 | 8.39±0.43 |
| 50-59 | 414 | 124.60±67.10 | 95.81±10.85 | 8.58±0.49 |
| 60-80 | 198 | 126.06±65.89 | 98.96±11.88 | 8.63±0.48 |
|  | **Jilin** | | |  |
| **Female** | **N** | **TG(mg/dl)** | **GLU(mg/dl)** | **TyG index** |
| Age groups |  |  |  |  |
| 20-29 | 81 | 90.43±44.98 | 90.97±7.39 | 8.23±0.43^a^ |
| 30-39 | 300 | 91.43±46.64 | 92.57±7.45 | 8.25±0.44 |
| 40-49 | 378 | 109.89±63.85 | 95.57±9.53 | 8.43±0.51 |
| 50-59 | 269 | 129.20±65.91 | 98.94±12.09 | 8.65±0.48 |
| 60-80 | 120 | 132.12±63.69 | 102.33±11.63 | 8.71±0.48 |

Data were presented as mean ± standard deviation (SD).

Different superscripts indicated significant differences between groups (*P* < 0.05, *P* value was corrected by Bonferroni method).
